# Using *In Situ* Symbiotic Seed Germination to Restore Over-collected Medicinal Orchids in Southwest China

**DOI:** 10.3389/fpls.2017.00888

**Published:** 2017-06-07

**Authors:** Shi-Cheng Shao, Kevin S. Burgess, Jennifer M. Cruse-Sanders, Qiang Liu, Xu-Li Fan, Hui Huang, Jiang-Yun Gao

**Affiliations:** ^1^Center for Integrative Conservation, Xishuangbanna Tropical Botanical Garden, Chinese Academy of SciencesMengla, China; ^2^Department of Biology, Columbus State University, ColumbusGA, United States; ^3^State Botanical Garden of Georgia, University of Georgia, Athens, GAUnited States; ^4^Graduate University of Chinese Academy of SciencesBeijing, China; ^5^Laboratory of Ecology and Evolutionary Biology, State Key Laboratory for Conservation and Utilization of Bio-resources in Yunnan, Yunnan UniversityKunming, China

**Keywords:** restoration-friendly cultivation, compatible fungi, *in situ* symbiotic seed germination, medicinal orchid, orchid conservation, Xishuangbanna

## Abstract

Due to increasing demand for medicinal and horticultural uses, the Orchidaceae is in urgent need of innovative and novel propagation techniques that address both market demand and conservation. Traditionally, restoration techniques have been centered on *ex situ* asymbiotic or symbiotic seed germination techniques that are not cost-effective, have limited genetic potential and often result in low survival rates in the field. Here, we propose a novel *in situ* advanced restoration-friendly program for the endangered epiphytic orchid species *Dendrobium devonianum*, in which a series of *in situ* symbiotic seed germination trials base on conspecific fungal isolates were conducted at two sites in Yunnan Province, China. We found that percentage germination varied among treatments and locations; control treatments (no inoculum) did not germinate at both sites. We found that the optimal treatment, having the highest *in situ* seed germination rate (0.94-1.44%) with no significant variation among sites, supported a warm, moist and fixed site that allowed for light penetration. When accounting for seed density, percentage germination was highest (2.78-2.35%) at low densities and did not vary among locations for the treatment that supported optimal conditions. Similarly for the same treatment, seed germination ranged from 0.24 to 5.87% among seasons but also did vary among sites. This study reports on the cultivation and restoration of an endangered epiphytic orchid species by *in situ* symbiotic seed germination and is likely to have broad application to the horticulture and conservation of the Orchidaceae.

## Introduction

Achieving a balance between biodiversity conservation and market demand for medicinal plants has become an urgent task for orchid conservationists world-wide. Consideration of symbiosis is one of the key factors for the restoration of declining orchid populations. Orchid seeds cannot germinate under natural conditions unless they are colonized with compatible mycorrhizal fungi that supply seeds and young plants with carbon and inorganic nutrients (Rasmussen, 1995). To overcome this hurdle, and to restore over-collected orchid populations in the wild, a number of *ex situ* tissue culture techniques have historically been used for the propagation and subsequent planting of orchid propagules within their natural distributions. These include: the generation of clones (*Ipsea malabarica*: Martin, 2003), the culturing of protocorms inoculated with mycorrhizal fungi (*Habenaria radiata*: Takahashi et al., 2008), the germination of seeds without symbiotic mycorrhizae (*Paphiopedilum wardii*: Zeng et al., 2012), and the use of soil to facilitate symbiotic seed germination (*Spiranthes brevilabris*: Stewart et al., 2003). While many *ex situ* technqiues have proven effective for the generation of orchid germplasm to facilitate resortation efforts, techniques that use low cost *in situ* methods are preferred for the maintaince and promotion of genetic diversity within remaining orchid populations.

Typically, the *Dendrobium* industry in China uses asymbiotic tissue culture to meet large-scale market demand for medicinal products where an abundance of seedlings can be generated over short periods of time and under controlled conditions. Large scale, *ex situ* production, however, often requires techniques that are labor intensive to acclimatize and transplant propagules; involve costly greenhouse and watering facilities; and use chemical pesticides and fertilizers. Furthermore, medicinal orchid products that are cultivated under such conditions are usually considered to be of low quality by consumers and in turn there is increasing market demand for naturally grown materials. Such attitudes have driven unprecedented collection pressures on wild populations that are unsustainable and have resulted in the endangerment of numerous orchid species (Li et al., 2008; Zeng et al., 2012; Khamchatra N. et al., 2016). *Ex situ* generation of plant material rarely accounts for the maintenance of a genetic diverse propagule pool for the restoration of over-exploited natural populations. To overcome the fact that seedling sources are often a bottleneck in the process of commercial orchid cultivation as well as the preservation of genetically diverse natural orchid populations, a conservation strategy that off-sets wild collection and market demands with restoration techniques is urgently needed.

The genus *Dendrobium* consists of approximately 1500 species, with 78 species existing in China (Chen et al., 2011). Many species of *Dendrobium* are used in traditional Chinese medicine to treat chronic diseases, and many populations of these species have been exploited to the point of local extirpation (Liu et al., 2014). Recently, Liu et al. (2013, 2014) proposed a new concept – restoration-friendly cultivation – as an unconventional method for the reintroduction of *D. officinale* seedlings within the species’ natural distribution in southwest China. While their technique provided a commercially viable restoration strategy that produced sustainable numbers of plants growing on the trunks of host trees, or in nursery beds, plant material generated *ex situ* encumbered substantial costs and labor. Furthermore, the fact that *Dendrobium* species in general are slow growers and highly sensitive to changes of microhabitats or locations remains a substantial barrier to natural regeneration and growth in the wild. Although it has been shown that some seedlings of terrestrial orchids have low survival rates, slow growth and delayed flowering after transplanting (Shimura and Koda, 2005; Stewart and Kane, 2006; Wu et al., 2010), plantlets co-cultured with compatible fungi grow faster with high survival rates (Batty et al., 2006; Johnson et al., 2007). Notably, the presence of compatible symbiotic fungi in orchid seedlings results in better adaptation to the environment leading to higher survivorship and faster growth than asymbiotic seedlings (Stewart et al., 2003; Johnson et al., 2007). Based on a comprehensive understanding of orchid mycorrhizae, it should be possible to utilize orchid mycorrhizal fungi to develop artificial propagation *in situ* to facilitate the reintroduction of selected endangered species to wild habitats (Luo et al., 2003).

To address the needs of the Chinese medicinal orchid industry as well as the needs of orchid conservation and restoration management, we assessed “*advanced* restoration-friendly” cultivation techniques in *D. devonianum*. Specifically, we conducted a series of *in situ* symbiotic seed germination trials using conspecific fungal isolates to address the following questions: (1) Do seeds sown *in situ* with symbiotic mycorrhizal fungus have higher germination rates compared to seeds sown without? (2) Does seed germination rate vary among microclimatic conditions and location? (3) Does seed density at the time of sowing influence seed germination rate?, and 4) Does the season in which seeds are sown influence germination rate? Ultimately, we hope to determine if *in situ* symbiotic seed germination can be used to conserve the endangered species *D. devonianum* by concomitantly rebuilding populations in the species’ natural range and meeting the demand of industry for cultivated medicinal orchid materials. This study is the first to report on the cultivation and restoration of an endangered epiphytic orchid species by *in situ* symbiotic seed germination, and is likely to have broad application to the horticulture and conservation of orchids.

## Materials and Methods

### Study Sites

Given that *D. devonianum* typically resides as an epiphyte on tree trunks of *Camellia* spp., our study sites were centered on two traditional tea gardens that contained numerous mature specimens of *Camellia sinensis* var. *assamica* in Xishuangbanna Dai Autonomous Prefecture, Yunnan Province, China. In this area, annual precipitation due to the tropical monsoon climate (rainy season occurs from May to October) ranges between 1,400 and 1,800 mm/yr. The rainy season is followed by a misty cool season from November to February, both March and April are the dry hot season. Annual average temperature and average relative humidity at both sites range between 18–20°C and 86–89%, respectively. Longpa traditional tea garden in Jinghong city (21°59′05′′ N; 101°05′07′′ E; altitude, 1150 m) is contiguous with tropical montane evergreen broad-leaved forests. Most of the tea trees had been cultivated for more than 200 years and they have an average height of 2 m. The remaining native shade trees include *Schima wallichii*, *Nephelium chryseum*, *Castanopsis echidnocarpa*, *Machilus gamblei*, *Alseodaphne andersonii*, *Meliosma arnottiana*, and *Macaranga indica*. Yiwu traditional tea garden is located in Mengla county (21°56′18′′ N, 101°28′13′′ E; altitude, 1200 m) and was established in the 1970s. The main shade trees are *Lauraceae* sp. and *Ehretia thyrsiflora*. Tea trees at the Longpa site were on average larger than those at Yiwu site, and the two sites are 90 km apart.

### Plant Species and Fungus

The study species, *D. devonianum*, is an epiphytic orchid widely distributed in the subtropical and tropical areas of SE Asia, including southwest China at altitudes from 1100 to 1900 m. In Xishuangbanna, it flowers from April to May and seeds mature in the following March (Gao et al., 2014). In July of 2012, a total of five naturally occurring protocorms of *D. devonianum* were obtained near maternal plants of *D. devonianum* growing in the wild, and one fungal strain (*Tulasnella* sp.) was isolated from each of the five protocorms. We experimentally tested that the fungus was capable of significantly promoting seed germination and subsequent seedling development (Huang et al., unpublished data). The strain was deposited in Mycological Herbarium, Institute of Microbiology, Chinese Academy of Sciences (HMAS^1^: accession number CGMCC No. 9551). The ITS sequence was submitted to GenBank database in the National Center for Biotechnology Information (NCBI: accession number KM226996.1).

### Seed Collection, Viability, and Storage

Mature, undehisced seed capsules of *D. devonianum* formed by natural pollination were collected in April 2014 in Tengchong county, Yunnan Province, within the natural range of the species. Seed capsules were sterilized in 75% (v/v) ethanol for 2 min and dried at room temperature before being opened with a scalpel under sterile conditions. The extracted seeds were transferred to airtight glass containers with calcium chloride anhydrous. After 4 days, the seeds were stored in a glass vial at -20°C for long-term preservation. Seed viability was 79.6% based on an assessment of approximately 300 seeds using a tetrazolium test (TTC).

### Fungal Inoculum and Suspension Composed of Seed and Mycelium

To ensure that *in situ* seed germination rates observed in this study are the result of the *Tulasnella* sp. inoculum that was isolated and applied in this study (as opposed to *in situ* contamination), *in vitro* symbiotic seed germination trials were conducted confirming the compatibility, efficacy and specification for promoting seed germination in *D. devonianum* (unpublished data). Subsequently the fungus was cultured in 10-50 ml conical flasks (500 ml) with 100 ml sterilized potato dextrose broth (PDB) placed on a shaker (ZQZY-A, Shanghai Zhichu Instruments Co., Ltd, China) at 150 rpm and 25°C for 7 days. The fermented mycelia were filtered with medical gauze and washed with sterile distilled water to avoid dextrose residue. One gram of fresh mycelium was homogenized in 50 ml sterile ddH_2_O by a sterile homogenizer. Seeds were added to this mixture and shaken to obtain a homogeneous liquid suspension. Prior to each round of seed sowing, seed density/ml of suspension was calculated by placing 200 μl of suspension on a glass slide and counting the total number of seeds/slide. This procedure was replicated three times per suspension and the mean was calculated.

### Sowing Methods and Treatments

Once the liquid suspension was prepared, seeds were sown on the trunks of *Camellia assamica* trees: in each case, 4 ml of liquid seed suspension was applied using a medical syringe. Because orchid seeds are easily flushed by rain due to their small size and require a moist, fertile inoculated substrate for seed germination, one of the goals of our study was to evaluate a number of plausible scenarios that might influence epiphytic *in situ* seed germination. The treatments were (1) Sphagnum: seed suspension applied between the trunk and a sphagnum wrap; (2) Trunk: seed suspension applied directly on the trunk; (3) Dung: seed suspension mixed with cow dung and applied directly to the trunk; (4) Plastic Wrap: seed suspension applied between the tree trunk and a plastic wrap; (5) Plastic Wrap + packet: seed suspension sealed in nylon packets attached to trunks using Plastic Wrap; (6) Control: seed suspensions containing seeds mixed in 0.1% sterile agar suspension without fungal inoculum. To ensure the liquid seed suspension remained fresh over time and was applied across a wide range of microhabitats on each tree, we randomly applied each treatment (∼8.5 treatments/tree) to approximately 200 trees that were randomly selected at the Longpa site (*N* = 1748). To evaluate the effect of location for treatments that showed the highest germination rates at the Longpa site, percentage germination was also assessed at the Yiwu site for treatments (4)–(6). Each treatment was also randomly applied to approximately 200 randomly selected trees (∼6 treatments/tree; *N* = 1215).

### Statistical Analysis of Seed Germination Rate

After 3 months, the number of seedlings for each treatment was counted and the percentage germination was calculated as (the number of seedlings/the number of viable seeds sown) × 100. Because percentage germination data (arcsine square root transformed) did not meet the assumptions of normality associated with the residuals of a one-way analysis of variance (ANOVA) model, we conducted non-parametric comparisons for each pair of treatments using Wilcoxon/Kruskal–Wallis tests (Rank Sums) among all six treatments. A number of sources of variation were evaluated. These include: (1) treatment, (2) seed density at time of sowing and (3) sowing season (rainy season [May to September], misty cool season [October to February], and dry hot season [March to April], and (4) location. Given a number of practical limitations associated with our study, such as two sites spanning multiple seasons, our design did not facilitate the testing of interaction terms among all treatments, densities, and seasons (lost DF in all cases); not all terms were represented in all categories. A one-way Chi-square approximation analysis for location as a source of variation was restricted to treatments (4)–(6); all three treatments were represented at both sites. In addition, and for the same reasons, we restricted our analysis of density of seeds sowed and sowing season as sources of variation to treatments (4) and (5). Here, we used a Wilcoxon/Kruskal-Wallis (Rank Sums) non-parametric comparison for each pair of terms in the model that included a treatment × density and a treatment × season analysis. For treatment (4) and (5), we analyzed three seed densities (51-100; 351-400; 501-550). All statistical analyses were conducted using JMP 10.

## Results

### Treatments

Overall, seed germination rates for the 2963 replicates conducted in this study across both sites varied from 0 to 83.01% (mean ± SE: 0.56 ± 0.10%) in *Dendrobium devonianum*. Within sites, mean germination rate was 0.59 ± 0.09% (mean ± SE) and 0.51 ± 0.10% at Longpa (*N* = 1748) and Yiwu sites (*N* = 1215), respectively.

**FIGURE 1:**
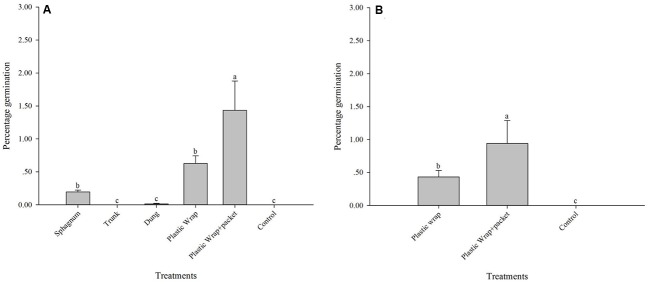
*In situ* symbiotic seed germination for *Dendrobium devonianum* at two study locations, Longpa and Yiwu, Yunnan, China. Letters above bars indicate significant differences in mean percentage germination. **(A)** *In situ* seed germination for the six treatments evaluated at the Longpa site; **(B)** *In situ* seed germination for the three treatments tested at the Yiwu site.

Significant differences among the treatments were found at the Longpa site (*X*^2^ = 90.11; *DF* = 5, *p* < 0.0001): the treatment (5) Plastic Wrap + packet (1.44 ± 0.44%; *N* = 216) had significantly higher percentage germination than the treatment (4) Plastic Wrap (0.63 ± 0.12%; *N* = 1075), and the treatment (1) Sphagnum (0.20 ± 0.03%; *N* = 229). The treatment (2) Trunk (mean = 0%; *N* = 103), treatment (3) Dung (0.0001 ± 0.000001%; *N* = 75), and treatment (6) Control (mean = 0%; *N* = 50) had significantly lower percentages of germination than all other treatments (**Figure 1A** and **Table 1**). Similar significant differences among the three treatments that were tested at that Yiwu site were found (*X*^2^ = 44.87; *DF* = 2, *p* < 0.0001): the treatment (5) Plastic Wrap + packet (0.94 ± 0.35%; *N* = 221) had significantly higher percentage germination than the treatment (4) Plastic Wrap (0.43 ± 0.10%; *N* = 947) (**Figure 1B** and **Table 1**). Similar to the results found at the Longpa site, both of treatment (4) Plastic Wrap and (5) Plastic Wrap + packet at the Yiwu site had significantly higher germination than treatment (6) Control (mean = 0; *N* = 47).

**Table 1:** Pairwise comparison of the effect of six treatments on seed germination in *Dendrobium devonianum* at the Longpa site and three treatments at the Yiwu site, both in Yunnan Province, China.

Location	Treatment	Sphagnum	Trunk	Dung	Plastic Wrap	Plastic Wrap and packet
Longpa	Trunk	-6.02770ˆ***	–			
	Dung	4.65704ˆ***	-1.65391	–		
	Plastic Wrap	1.52126	-5.17609ˆ***	3.91815ˆ***	–	
	Plastic Wrap + packet	-2.36149ˆ*	-7.13776ˆ***	5.70795ˆ***	4.53383ˆ***	–
	Control	4.28366ˆ***	–	1.14775	3.62376ˆ**	5.11186ˆ**
Yiwu	Plastic Wrap + packet				5.678244ˆ***	–
	Control				3.047632ˆ**	4.654356ˆ***

*Astericies indicate level of signification associated with each Chi-square statistic (^∗∗∗^*P* < 0.0001; ^∗∗^*P* < 0.01; ^∗^*P* < 0.05)*.

When the effect of location was compared directly, germination rate was significantly higher at Longpa site compared to the Yiwu site for treatment (4) Plastic Wrap (*X*^2^ = 9.99; *DF* = 1, *P* < 0.01). No difference in germination rates between the Longpa and Yiwu sites was found for treatment (5) Plastic Wrap + packet (*X*^2^ = 1.18; *DF* = 1, *P* = 0.28).

### Effect of Seed Density on Treatments

**FIGURE 2:**
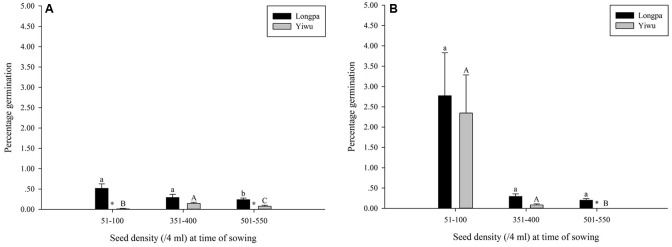
The effect of seed density at time of sowing for **(A)** Plastic Wrap and **(B)** Plastic Wrap + packet treatments on *in situ* symbiotic seed germination for *D. devonianum* at Longpa and Yiwu, Yunnan, China. Significant differences among seed densities are indicated by letters. Astericies show significant differences among sites.

Percentage germination differed significantly for seed density at time of sowing for the two treatments analyzed in each site. For treatment (4) Plastic Wrap, seed germination at the Longpa site was significantly lower for the highest seed density (0.24 ± 0.04%) compared to seeds sown at lower densities that ranged from 0.29% (*SE* = 0.08%) to 0.52% (*SE* = 0.11%) which did not differ significantly from each other (**Figure 2A** and **Table 2**). Seed germination for treatment (4) at the Yiwu site, was significantly higher for the medium seed densities (0.15 ± 0.03%) compared to seeds sown at lower (0.01 ± 0.01%) and higher (0.08 ± 0.03%) densities which were also significantly different from each other (**Figure 2** and **Table 2**). For treatment (5) Plastic Wrap + packet, seed germination at the Longpa site did not differ significantly among low (2.78 ± 1.06%), intermediate (0.30 ± 0.06%) and high (0.20 ± 0.04%) seed densities (**Figure 2** and **Table 2**). Seed germination at the Yiwu site, was significantly higher for the lowest (2.35 ± 0.94%) and intermediate (0.08 ± 0.03%) seed densities compared to seeds sown at the highest (mean = 0) density but did not differ significantly from each other (**Figure 2B** and **Table 2**).

**Table 2:** The effect of two seed sowing densities on seed germination in *D. devonianum* for two treatments (Plastic Wrap and Plastic Wrap + packet) at the Longpa and Yiwu study sites, Yunnan, China.

Location	Treatment	Density	51-100	351-400
Longpa	Plastic Wrap	351-400	0.65925	–
		501-550	-3.47137ˆ***	-2.33562ˆ*
	Plastic Wrap + packet	351-400	-0.730583	–
		501-550	-0.05296	-0.400359
Yiwu	Plastic Wrap	351-400	8.67301ˆ***	–
		501-550	-6.02305ˆ***	2.27328ˆ*
	Plastic Wrap + packet	351-400	-0.36000	–
		501-550	2.89915ˆ**	2.99688ˆ**

*Astericies indicate significant differences among all pairwise comparisons based on a Chi-squared test (^∗∗∗^*P* < 0.0001; ^∗∗^*P* < 0.01; ^∗^*P* < 0.05)*.

Significant differences among sowing densities were found for the two treatments analyzed in each site. For treatment (4) Plastic Wrap, seed germination at the Longpa site was significantly higher at low (*X*^2^ = 41.35; *DF* = 1, *P* < 0.0001) and high (*X*^2^ = 9.67; *DF* = 1, *P* = 0.0019) densities compared to the Yiwu site. Germination did not differ between sites for medium densities (*X*^2^ = 1.99; *DF* = 1, *P* = 0.1584) (**Figure 2**). When the effect of location for treatment (5) Plastic Wrap + packet was compared directly, germination rate was significantly higher at the Longpa site compared to Yiwu site for the highest density (*X*^2^ = 18.72; *DF* = 1, *P* < 0.0001) but the two sites did not differ significantly from each other at the lowest (*X*^2^ = 0.74; *DF* = 1, *P* = 0.39) and intermediate (*X*^2^ = 1.13; *DF* = 1, *P* = 0.29) densities (**Figure 2**).

### Effect of Sowing Season on Treatments

**FIGURE 3:**
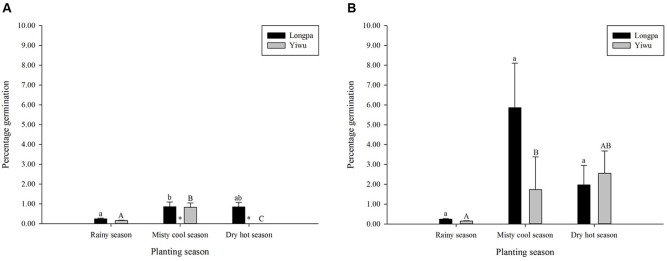
The effect of sowing season on *in situ* symbiotic seed germination for *D. devonianum* at two locations by **(A)** Plastic Wrap and **(B)** Plastic Wrap + packet treatments. Letters indicate significant differences among seasons and astericies show significant differences among Longpa and Yiwu sites in Yunnan, China.

Percentage germination differed significantly for sowing seasons for the two treatments analyzed in each site. For treatment (4) Plastic Wrap, seed germination at the Longpa site was significantly lower for the rainy season (0.25 ± 0.04%) compared to seeds sown at misty cool season (0.85 ± 0.24%) but neither differed significantly from the dry hot season (0.85 ± 0.23%) (**Figure 3A** and **Table 3**). Seed germination at the Yiwu site, was significantly higher for the misty cool season (0.83 ± 0.22%) compared to seeds sown during the rainy season (0.16 ± 0.02%) and the dry hot season (mean = 0) which were significantly different from each other (**Figure 3** and **Table 3**). For treatment (5) Plastic Wrap + packet, seed germination at the Longpa site did not differ significantly among the rainy (0.24 ± 0.03%), misty cool (5.87 ± 2.24%), and dry hot (1.97 ± 0.98%) seasons (**Figure 3B** and **Table 3**). Seed germination at the Yiwu site, was significantly lower for the rainy season (0.147 ± 0.02%) when compared to the misty cool season (1.74 ± 1.65%), although having the highest seed germination rates the dry hot season (2.55 ± 1.13%) was not significantly from either (**Figure 3B** and **Table 3**).

**Table 3:** Pairwise comparison of the effect of three sowing seasons on seed germination in *D. devonianum* for Plastic Wrap and Plastic Wrap + packet treatments at the Longpa and Yiwu sites, Yunnan, China.

Location	Treatment	Season	Rainy season	Misty cool season
Longpa	Plastic Wrap	Misty cool season	-3.76424ˆ**	–
		Dry hot season	-0.74519	1.89164
	Plastic Wrap + packet	Misty cool season	0.505016	–
		Dry hot season	-0.303257	-0.606977
Yiwu	Plastic Wrap	Misty cool season	-6.86380ˆ***	–
		Dry hot season	-6.76209ˆ***	-3.32583^∗∗^
	Plastic Wrap + packet	Misty cool season	-2.20663ˆ*	–
		Dry hot season	-0.08124	1.5862

*Chi-squared statistic and the associated significance (^∗∗∗^*P* < 0.0001; ^∗∗^*P* < 0.01; ^∗^*P*< 0.05) are listed for each pairwise comparison.*

Significant differences among the two sites were found for the sowing seasons analyzed in each site. For treatment (4) Plastic Wrap, seed germination at the Longpa site was significantly higher during the misty cool season (*X*^2^ = 7.13; *DF* = 1, *P* = 0.0076) and the dry hot season (*X*^2^ = 28.73; *DF* = 1, *P* < 0.001) compared to the Yiwu site. Germination rates did not differ significantly between sites for the rainy season (*X*^2^ = 0.064; *DF* = 1, *P* = 0.8008) (**Figure 3A**). When the effect of location for treatment (5) Plastic Wrap + packet was compared directly, germination rate did not differ significantly among sites for the rainy season (*X*^2^ = 0.4908; *DF* = 1, *P* = 0.4836), misty cool season (*X*^2^ = 3.4451; *DF* = 1, *P* = 0.0634), and dry hot season (*X*^2^ = 0.0008; *DF* = 1, *P* = 0.9768) (**Figure 3B**).

## Discussion

### The Role of Inoculum on *In Situ* Seed Germination

It is well known that the seeds of orchid species require compatible mycorrhizal fungi for germination to occur in nature, and symbiotic fungi have widely been shown to promote seed germination and facilitate seedling establishment in both terrestrial and epiphytic orchids (Zelmer and Currah, 1997; Liu H. et al., 2010). For some orchid species, however, asymbiotic seed germination can also result in the rapid production of seedlings *ex situ* (Johnson et al., 2007; Zeng et al., 2012). Although asymbiotic seedlings can be transplanted successfully in the wild, they often grow slower, and have higher mortality rates, and flower phenology is often delayed (Shimura and Koda, 2005; Batty et al., 2006; Stewart and Kane, 2006; Wu et al., 2010). As a result, the reintroduction of symbiotic seedlings into natural habitats that were generated *ex situ* has been a primary component of restoration plans for many orchid species to date. This technique, however, is often expensive and labor intensive and is thus of limited conservation value on a wide scale. Furthermore, restored populations often lack the genetic variation necessary for local adaptation and evolutionary potential (Zhou and Gao, 2016). In this study we tested whether the presence or absence of a fungal inoculate would influence *in situ* symbiotic seed germination. We proposed the question, can seeds germinate directly in the field and develop into artificial populations without laboratory facilities, artificial transplantation and acclimatization from *ex situ* conditions? As expected, no protcorms or seedlings emerged after 3 months in Control tests (those lacking fungi) suggesting that protocorms or seedlings can be formed only with the presence of symbiotic fungi (**Figures 1A,B**). Critical to seed germination and seedling success, compatible fungi provide carbohydrates, vitamins, and growth factors that appear, based on our results, imperative for the culture of orchids *in situ*.

**FIGURE 4:**
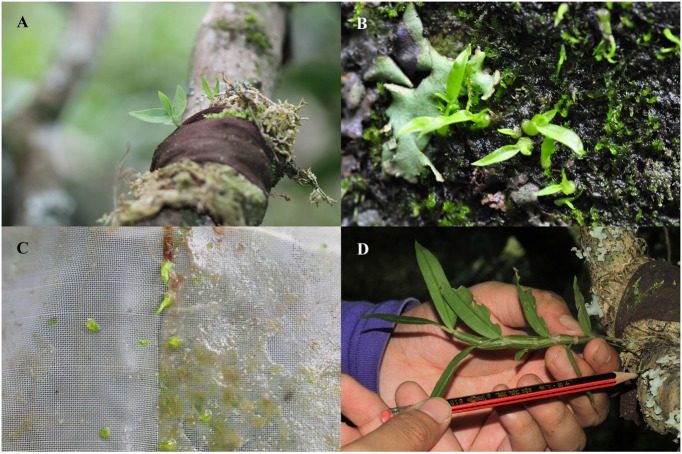
Results of *in situ* symbiotic seed germination for *D. devonianum* in Yunnan, China. **(A)** Two-month old seedlings using the Sphagnum treatment in Longpa; **(B)** Seedlings formed by the Plastic Wrap treatments in Longpa 45 days after sowing; **(C)** Seedlings formed by the Plastic Wrap + packet treatments in Yiwu 30 days after sowing; **(D)** Twelve-month old seedlings grew to 10 cm after sowing using the Sphagnum treatment in the Longpa site, Yunnan, China.

Mycobiont specificity, however, between closely related orchid species may require special consideration for the *in situ* symbiotic propagation and conservation of orchid species (Stewart and Kane, 2007). Numerous studies on symbiotic seed germination in orchids using fungi isolated from roots, regardless of host specificity, can result in the successful production of rhizoids, protocorms, and seedlings (Stewart and Zettler, 2002; Nontachaiyapoom et al., 2011; Tan et al., 2014). Although we did not test alternative inoculum in our study, *D. devonianum* seeds inoculated with host mycorrhizae germinated 30-45 days after sowing and further formed seedlings at 60 days (**Figures 4A–C**), indicating that use of the fungal symbiont isolated from orchids in the natural population may lead to successful germination and development of seedling in the *in situ* restored population. Interestingly, Sebastian et al. (2014) used five fungal strains from the corresponding host of *Aa achalensis* to promote seed germination and found that seed germination depended on the strains used. Varying results have also been shown in *Gavilea australis* where percentage seed germination varied depending on the origin of the mycorrhizal fungi isolated from the host (roots or protocorm) or conspecific vs. heterospecific species (Fracchia et al., 2014). Based on our results, as well as those of previous studies, it seems likely that *advanced* restoration-friendly techniques should include the screening of compatible mycorrhizal fungi (as in Luo et al., 2003) from corresponding host protocorms to promote *in situ* seed germination.

To provide a basic understanding of the biology of orchids and how it contributes to their conservation, knowledge of the origin of mycorrhizal fungi and its impact on orchid growth is another primary consideration for *advanced* restoration-friendly techniques that promote *in situ* seed germination (Batty et al., 2001; Liu H.X. et al., 2010). Although many fungal strains have been shown to promote orchid seed germination, it is also important to assess compatibility between the origin of the fungal isolate and orchid seedling development (Nontachaiyapoom et al., 2011). Although the fungi from protocorms and roots are often of the same tullasnelloid genus, many orchids require a narrower range of fungi at the protocorm stage than that required for germination (Rasmussen, 2002). For example, although Stewart and Kane (2006) used six fungal mycobionts to promote seed germination, protocorms in many cases only survived to early developmental stages. As a result, numerous studies have obtained fungal isolates from corresponding orchid protocorms formed by *in situ* and *ex situ* baiting and not plants roots (Rasmussen and Whigham, 1993; Wang et al., 2011; Zi et al., 2014). In addition to the reasons listed above and in the absence of specific knowledge of species associated with the orchids in natural populations, we believe that the optimal source of fungal mycobionts for *in situ* symbiotic germination is from the protocorms of the corresponding host because development of the orchid seedlings is more sensitive to host specificity than what is required for germination. For the purpose of the conservation of endangered or declining orchid species as well as meeting the demands of commercial production, mycorrhizal association and specificity are factors that cannot be neglected and require future research.

### The Best Sowing Methods

Historically, attempts to initiate germination in epiphytic orchids *in situ* have involved the placement of seeds onto organic substances such as sphagnum moss, bark, or leaf mold, which often prove to be unsuccessful (Kauth et al., 2008). Sphagnum moss, however, which allows for light penetration and water retention, has often been used for seedling cultivation, acclimatization in greenhouse settings or for seedling reintroduction to prevent desiccation in the field (Zettler et al., 2007; Valadares et al., 2012; Zeng et al., 2012; Khamchatra N.M. et al., 2016). To evaluate the role of humidity on *in situ* seed germination that would mimic moisture and light conditions in sealed petri dishes in the laboratory or on sphagnum substrate in the greenhouse, we tested a Plastic Wrap treatment for germination trials in the field. We also tested a Plastic Wrap + packet treatment that we hypothesized would not only maintain optimal humidity and light levels but would also keep seeds in a fixed site. Although both treatments showed significantly higher germination compared to all the organic treatments in our study, the highest germination rates were found for the Plastic Wrap + packet treatment, a trend that was consistent across both our study sites (**Table 1** and **Figures 1, 4**). The water retention capabilities of this treatment, coupled with the fact that our seeds were not washed or blown away over time, seems a likely cause for the success of this treatment. Many cultivation parameters, however, such as microclimatic conditions, seed density at time of sowing, and planting season, to name a few, may also be affecting seed germination of *D. devonianum* in field. Although our research is limited to *in situ* seed germination of this wild Chinese species, below we discuss some of the main sources of variation that may be contributing to our results and may have broader application to the Orchidaceae.

### Influence of Microclimate and Location

Germination for the two plastic treatments occurred at both sites but significant variation in germination among sites only occurred for the Plastic Wrap treatment and not for the Plastic Wrap + packet treatment. Thus it would appear that the conditions promoted by the Plastic Wrap + packet treatment buffers microclimatic variation among sites whereas our Plastic Wrap treatment seems to be more sensitive to unfavorable conditions. Previous studies have shown that seed germination may be influenced by locations that have distinct microhabitats which reflect differences in light, temperature, and humidity; all of which are probably indirectly related to seed germination rates by affecting seed viability or by influencing fungal growth or survival. For example, Batty et al. (2006) successfully propagated seedlings of *Caladenia arenicola* by *in situ* symbiotic seed germination but none of the seedlings survived summer dormancy, perhaps due in part to the lack of favorable microclimatic conditions necessary for the maintenance of seedlings and fungal vitality. Although the extant natural distribution of *D. devonianum* includes both our study sites and thus both locations were suitable for seed germination and growth, seed germination for our Plastic Wrap + packet treatment was less affected by microhabitat variation.

### The Effect of Seed Density at the Time of Sowing

We found that seed germination was significantly higher at low seed densities compared to germination at higher densities (**Table 2** and **Figure 2**). Although we are not aware of any previous studies on density-dependent processes associated with *in situ* seed germination in epiphytic orchids, symbiotic seed germination trials on OMA medium showed that the seeds of *D. devonianum* could not initiate germination with >1500 seeds in a 9 cm petri dish (unpublished data). *In vitro* symbiotic seed germination usually has <200 seeds per petri dish (Stewart and Zettler, 2002; Stewart et al., 2003; Zi et al., 2014) and seldom >500 seeds are used (Park and Lee, 2013). Based on our results it would appear that optimum seed density for *in situ* seed germination is approximately 50-100 seeds per 4 ml of liquid suspension at the time of sowing (**Figure 2**), a result that is concordant with most *ex situ* studies. Although the exact mechanism driving this threshold remains elusive, there is some evidence to suggest that seed germination rate is partly dependent on the seed density in suspension prior to application of our treatments because of increased carbon acquisitions driven by increasing volume of fungi in suspension. Additionally, the effect of seed density varied among sites for only the Plastic Wrap treatment, indicating that a density by microhabitat interaction may be also important, although given the statistical power of our dataset we were unable to test this interaction directly. It would appear that when microclimatic conditions promote a stable, moist, warm substrate (Plastic Wrap + packet treatment) seed germination is not only better but more consistent regardless of seed density at the time of sowing.

### Time of Sowing

Seed germination rate varied among seasons and trends varied among sites. Most surprising was that the highest seed germination was during the misty cool season followed by the dry hot season, both in stark contrast to the lowest rates which were generally found during the rainy season (**Figure 3**). Although we were unable to directly test the season × density interaction (which may also be a casual factor for our results) due to lost degrees of freedom, the most likely abiotic factor influencing *in situ* seed germination across seasons is the humidity level of the microhabitat, which can in turn affect germination success, subsequent protocorm development and survivorship. Because plantlets can not survive for more than several days without humidity around their roots, the absolute number of seedlings formed during the rainy season was much higher and the seedlings looked more fit compared to results found for other seasons (per obs., **Figure 4**). Additionally, the effect of season on seed germination varied among sites for only the Plastic Wrap treatments, indicating that a season and microhabitat interaction may be an important factor driving our results, although we were also unable to test this directly due to lack of statistical power in our dataset. It would appear that regardless of season, microclimatic conditions favoring a more stable moist and warm substrate (the Plastic Wrap + packet treatment) promote more consistent and better seed germination. Given that natural seed formation occurs from May to March and protocorms that are used for fungal isolation are typically found in July, we speculate that the ideal season for *in situ* seed sowing as an advanced cultivation-friendly technique at our sites is from November to January (the misty cool season).

### Concluding Remarks and Future Recommendations

Advanced restoration-friendly cultivation of orchids through *in situ* symbiotic seed germination can produce a large number of orchid seedlings quickly and easily at very low cost. For successful application, however, fungal origin and compatibility should be considered prior to sowing seeds of epiphytic orchid species in the wild. In addition, based on our research findings, we suggest that advanced *in situ* germination techniques include a secure sowing site that promotes a warm microhabitat with high and constant humidity yet allows light penetration to promote germination. It would also appear that treatments that promote such conditions (such as our Plastic Wrap + packet treatment) will have high germination rates regardless of variation in microhabitat conditions or location, and variation associated with seed density, or the timing of planting (season), although geographic origin and potential ecological risks remain to be tested (Zettler et al., 2007). We hope that after 2 or 3 years of growth, fresh orchid stems (**Figure 4**) for use in the medicinal plant trade, produced through the cultivation techniques outlined in this paper, will have the same quality as their wild counterparts. Our results have enormous potential for the production and sale of locally produced materials while simultaneously easing collection pressures on native plant populations. Although our research is the only based on *in situ* seed germination of Chinese wild species, our study has broad application for the both commercial and conservation initiatives for the Orchidaceae.

## Author Contributions

SCS and JYG designed experiments. SCS, QL, XLF, HH, and JYG conducted experiments. SCS, KB, JCS, and JYG wrote the manuscript.

## Conflict of Interest Statement

The authors declare that the research was conducted in the absence of any commercial or financial relationships that could be construed as a potential conflict of interest.

## Footnotes

1 http://hmas.im.ac.cn
